# Chronic myelomonocytic leukemia primarily presenting as life‐threatening pericardial effusion, Eldoret, Kenya: A case report

**DOI:** 10.1002/ccr3.9048

**Published:** 2024-06-07

**Authors:** Victor M. Wauye, Evangeline Njiru, Angela K. Amadi, Mildred N. Hagembe, Gabriel Kigen

**Affiliations:** ^1^ Department of Internal Medicine, School of Medicine College of Health Sciences, Moi University Eldoret Kenya; ^2^ Department of Internal Medicine Moi Teaching and Referral Hospital Eldoret Kenya; ^3^ Department of Pharmacology and Toxicology, School of Medicine College of Health Sciences, Moi University Eldoret Kenya

**Keywords:** ascites, chronic myelomonocytic leukemia, heart failure, pericardial effusion, pleural effusion

## Abstract

**Key Clinical Message:**

Chronic myelomonocytic leukemia, a rare case of hematological malignancy mainly affects the elderly and may present with life threatening pericardial effusion as an initial manifestation. High index of suspicion is hence key for early management.

**Abstract:**

We present a case of an 81‐year‐old African male who presented with progressive cough, respiratory distress and bilateral lower limb swelling, and was diagnosed with life‐threatening pericardial effusion resulting from chronic myelomonocytic leukemia following complete blood count, peripheral blood film, bone marrow aspirate with trephine biopsy, and flow cytometry studies.

## 1 INTRODUCTION

Chronic myelomonocytic leukemia (CMML) is a hematological malignancy characterized by both clinical and pathological features of myeloproliferative (MPN) and myelodysplastic (MDS) syndrome. It is evidenced by peripheral monocytosis and myeloid precursor cell dysplasia.^1^ According to 2016 World Health Organization (WHO) classification of myeloid neoplasms and acute leukemias, CMML is classified under the group of MDS/MPN neoplasms, together with juvenile myelomonocytic leukemia, atypical chronic myeloid leukemia (CML), and ring sideroblasts with thrombocytosis. This group also constitutes the MDS/MPN unclassified type.^2^, ^3^

CMML is relatively a rare disorder, with a current reported incidence of four cases per 100,000 persons/year.^4^ It mainly affects the elderly with a median age of 71–73 years. CMML presents heterogeneously, with clinical features ranging from constitutional symptoms, splenomegaly to cytopenias as well as extramedullary disease, but rarely causes life threatening extramedullary complications such as pericardial and pleural effusion as well as ascites.^5^, ^6^, ^7^ We report a case of an 82‐year‐old male patient with CMML who presented with pericardial effusion and acute heart failure (HF).

## 2 CASE PRESENTATION/EXAMINATION

An 82‐year‐old male patient was admitted to Moi Teaching and Referral Hospital, Eldoret on 1st April 2022 with progressive cough, difficulty in breathing and bilateral lower limb swelling for 4 weeks. He had a previous history of on and off cough since June 2021. The cough was mostly dry, but sometimes associated with whitish sputum. It was not variable by time of the day and was initially not associated with hotness of body, paroxysmal nocturnal dyspnoea, orthopnoea, easy fatiguability, or lower limb swelling. However, 4 weeks prior to admission, the cough became persistent and associated with difficulty in breathing, dyspnoea on mild exertion, progressive bilateral lower limb swelling, abdominal distension, and early satiety. He also reported unintentional weight loss, but no history of other known chronic illnesses such as diabetes mellitus, hypertension, asthma, chronic obstructive pulmonary disease, and tuberculosis.

On physical examination, he had bilateral non‐tender pitting oedema up to the knee level. His BP was 120/70 mmHg, PR 79 bpm, RR 18 bpm, and SPO_2_ 75% room air (which improved to 96% on 7 L of O_2_ via non‐rebreather mask), and temperature of 36.8°C. Cardiovascular exams revealed warm extremities, normal volume peripheral pulses, muffled heart sounds but no cardiac murmurs. Abdominal examination revealed nontender abdominal distension, hepatomegaly (4 cm below the costal margin), shifting dullness but no splenomegaly. Respiratory and central nervous systems were unremarkable.

## 3 METHODS (DIFFERENTIAL DIAGNOSIS, INVESTIGATIONS, AND TREATMENT)

Complete blood count revealed WBCs of 75.32 × 10^9^/L, absolute neutrophils: 50.56 × 10^9^/L (67.6%), monocytes: 18.50 × 10^9^/L (24.0%), lymphocytes: 4.76 × 10^9^/L (6.6%), Hb 12 g/dL, and platelets of 142 × 10^9^/L. NT‐proBNP was 4800 pg/mL (normal <125 pg/mL). Renal, liver and thyroid function tests were largely unremarkable. Blood culture yielded no growth, ruling out sepsis.

**FIGURE 1:**
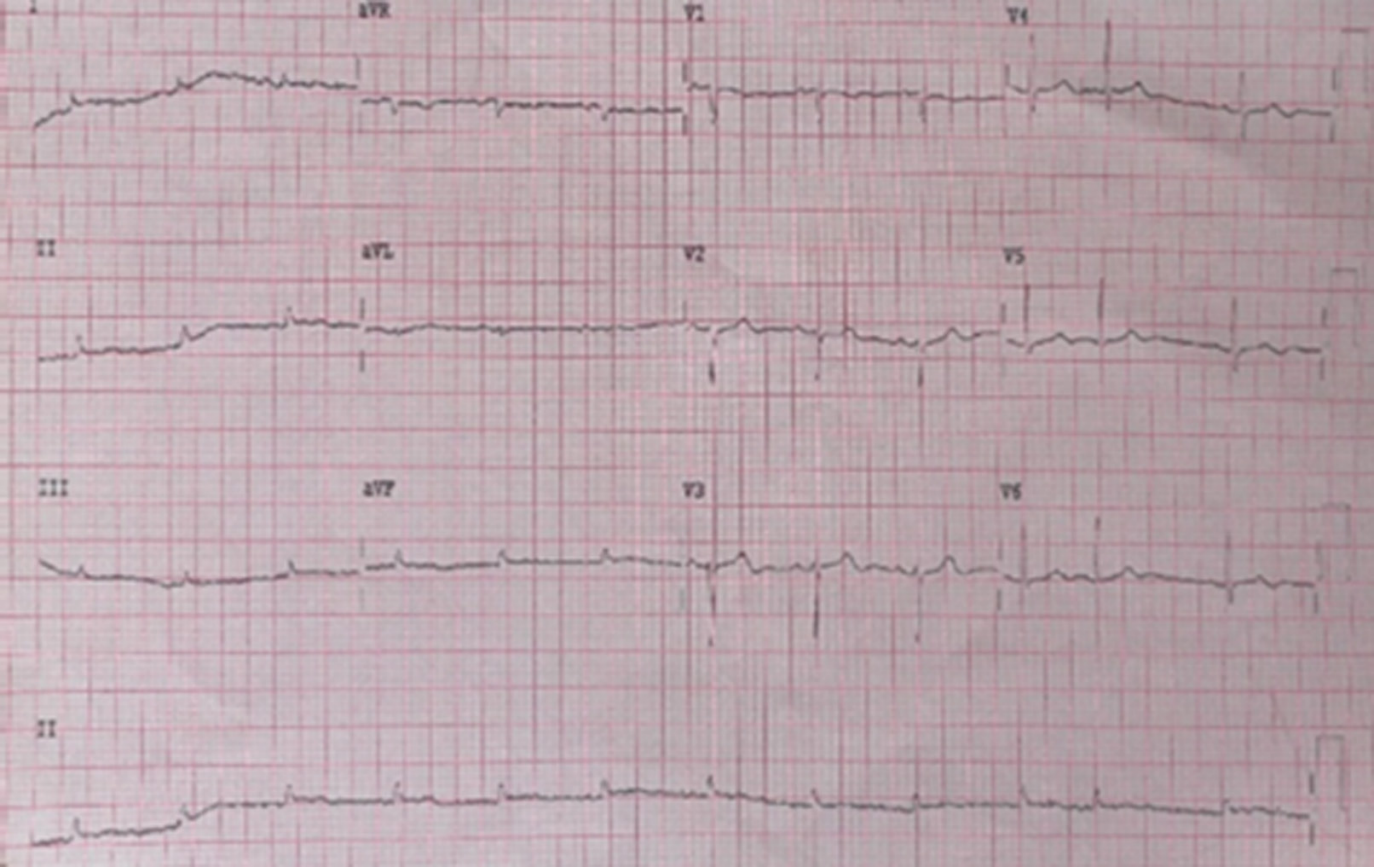
ECG image showing low voltage QRS complexes and electrical alternans.

Electrocardiography showed low voltage QRS complexes with electrical alternans (Figure 1). Echocardiography showed large circumferential pericardial effusion (3.0 cm), swinging heart, RA/RV free wall collapse with evidence of pulmonary hypertension (RVSP of 43 mmHg), dilated non‐collapsing IVC with normal left and right ventricular function.

**FIGURE 2:**
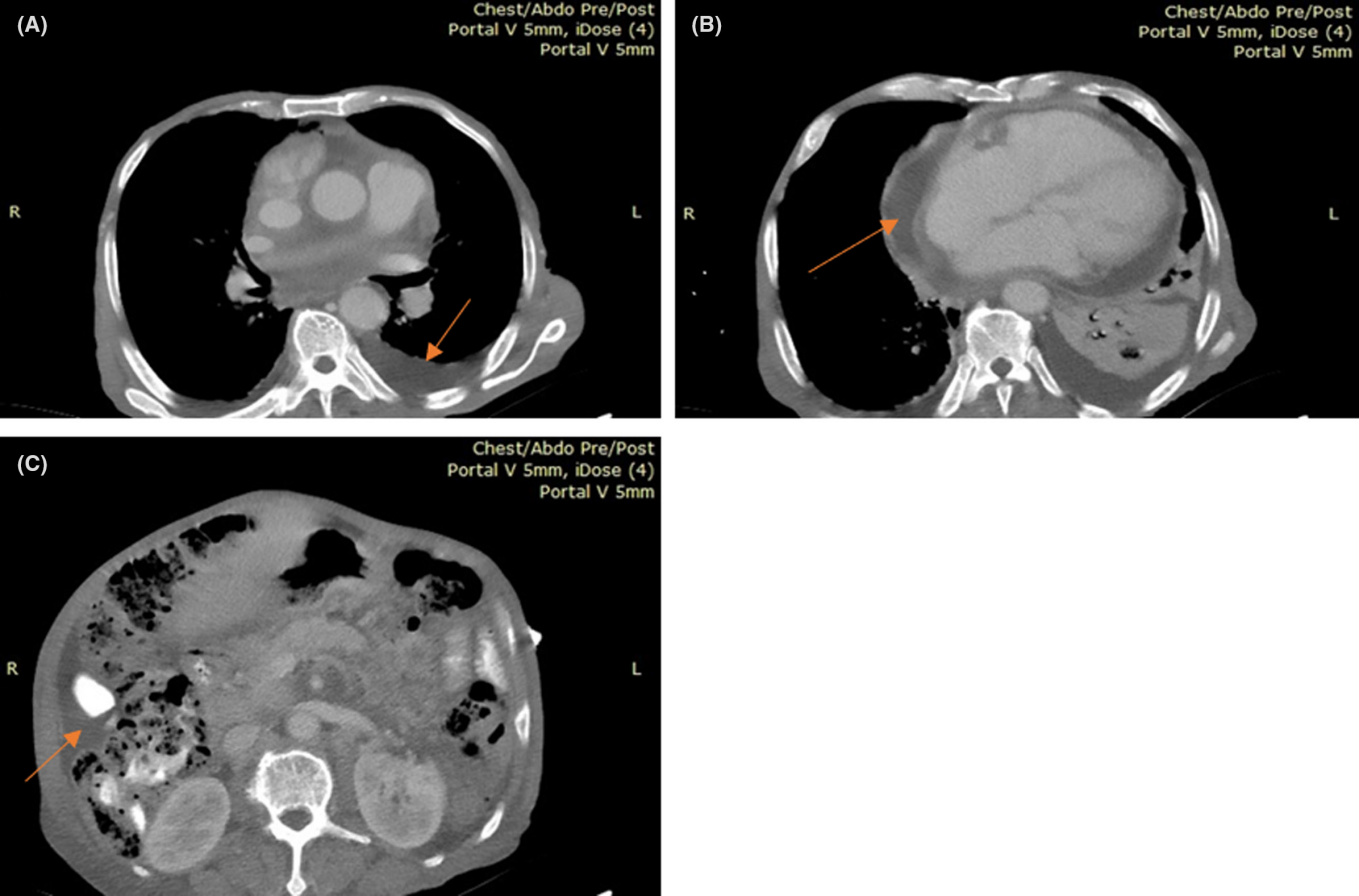
Chest/abdominal CT scan images showing pleural effusion (A), pericardial effusion (B), and ascites (C) pointed with the arrows.

Chest and abdominal CT scan showed pericardial and bilateral pleural effusion, and moderate ascites with multiple para‐aortic and mesenteric nodes (Figure 2). Peripheral blood film showed monocytosis, and left shift with less than 1% blasts. Bone marrow aspirate with trephine biopsy showed hypercellular marrow with myeloid hyperplasia but no excess blasts, while flow cytometry was 94% positive for CD14, with remarkable monocyte population with aberrant CD123 (94%) and CD56 (83%), highly suggestive for CMML. Based on these findings, we arrived at a diagnosis of CMML presenting with acute HF due to life threatening pericardial effusion.

Pericardiocentesis with placement of pigtail catheter was done, draining a total of 1800mLs of haemorrhagic fluid (800mLs on day 1, 750mLs day 2, 150mLs day 3, and 100mLs day 4). This resulted in the relief of the patient's symptoms, including weaning from oxygen supplementation. Pleural fluid was exudative, with the total protein of 63.5 g/L against total serum protein of 74.7 g/L (pleural fluid: serum total protein ratio of 0.85). Both pericardial and pleural fluid culture and gram‐staining for acid‐fast bacteria were negative ruling out pericardial tuberculosis. The patient was therefore started on diuretics: frusemide and spironolactone for the HF, and hydroxyurea 1 g twice daily for the CMML.

## 4 CONCLUSION AND RESULTS (OUTCOME AND FOLLOW‐UP)

The patient clinically improved and was discharged from hospital on day 21, with the discharge WBC being 36.92 × 10^9^/L, neutrophils 18.58 × 10^9^/L (50.3%), monocytes 10.44 × 10^9^/L (28.3%), lymphocytes 7.02 × 10^9^/L (19.0%), Hb 10.6 g/dL and platelets 35 × 10^9^/L. He was scheduled for follow‐up at the MTRH haemato‐oncology clinic in 2 weeks, for which he returned and was doing well, with the WBC having reduced to 15.21 × 10^9^/L, neutrophils 8.35 × 10^9^/L, monocytes 3.44 × 10^9^/L, lymphocytes 2.66 × 10^9^/L, Hb 11.2 g/dL and platelets 100 × 10^9^/L. Further details on the laboratory findings are as shown in Data S1 document.

## 5 DISCUSSION

CMML is a rare hematological disorder, with most of the cases being asymptomatic at presentation. This makes the diagnosis of CMML challenging, especially in the low and middle‐income settings where diagnostic resources could be limited. Noting that CMML has an inherent risk of transforming to acute myeloid leukemia (AML) and this is associated with poorer outcomes,^8^, ^9^ high index of suspicion is therefore primal for early recognition and initiation of treatment. Our patient presented with acute HF from life‐threatening pericardial effusion, which is a much rare manifestation of CMML.^6^, ^7^ Therefore, the diagnosis of CMML as the primary cause of our patient's complaints could have been missed were it not for the attention paid to the pericardial effusion as well as white blood cell counts, which necessitated further investigations and early intervention, leading to prompt clinical improvement and relative early discharge from the hospital.

CMML diagnosis should be guided by high index of suspicion, supported by suggestive findings from complete blood count and bone marrow. Notably, clinical presentation of CMML is quite variable, ranging from being asymptomatic to constitutional symptoms such as malaise, weight loss and night sweats, as well as symptoms of specific cytopenias such as headache among those with anemia, bleeding tendencies among those with thrombocytopenia and recurrent infections due to white blood cell dysfunction.^10^ On the other hand, polyserositis, which refers to the inflammation of the serous membranes (pericardium, pleura and peritoneum) and formation of serous effusions is a rare presentation of CMML.^6^, ^11^, ^12^ Fauci et al. reported that polyserositis affected up to 20% of the cases.^13^ Of the 9723 cases analyzed by Kaur et al., only 0.4% (*n* = 40) had leukemic involvement of the serous membranes, with only one case being due to CMML.^14^ The most common site was pleural cavity (*n* = 30), followed by peritoneal cavity (*n* = 7), then pericardial cavity (*n* = 3). Therefore, our patient primarily presenting with life‐threatening pericardial effusion with acute HF was a much rarer case of CMML.

Pericardial effusion as an initial presentation of CMML is rare and has been reported in few case reports in literature,^7^, ^15^, ^16^, ^17^ reflecting the rarity of this presentation. Not detected early, pericardial effusion could lead to cardiac tamponade or acute HF as in our case. This is potentially fatal and is associated with high mortality. Markedly, malignant pericardial effusion carries a mortality risk of up to 80%.^18^, ^19^ Early detection and management of pericardial effusion and HF is therefore key in determining outcomes of patients with CMML. For symptomatic relief and to improve outcome, pericardial fluid drainage or pericardiocentesis is recommended.^20^ However specific treatment is directed towards the treatment of CMML with agents such as hydroxyurea for cytoreduction, and hypomethylating agents such as azacitidine and decitabine.^21^ Our patient responded well to pericardiocentesis which resulted in prompt symptomatic relief, and was later started on hydroxyurea before discharge.

CMML is classified as a MPN/MDS neoplasm under the 2016 WHO classification of myeloid neoplasms and acute leukemia.^2^, ^3^, ^21^ This was an improvement of the 2008 classication^22^; a change which improved the diagnosis of CMML by introducing the use of both absolute and relative monocytosis as well as exclusion of other MPN causes of monocytosis.^23^, ^24^ This would aid in reducing over diagnosis of CMML. Consequently, by the 2016 WHO diagnostic criteria, CMML is defined by: peripheral blood and bone marrow morphological findings of peripheral monocytosis of >1 × 10^9^/L, with monocytes comprising >10% of the total WBC persisting more than 3 months, <20% blasts in peripheral blood and bone marrow, and dysplasia affecting ≥1 myeloid cell lines.^5^, ^25^, ^26^ Further, exclusion of MPN that may present with reactive monocytosis such as CML, essential thrombocythemia (ET) and polycythaemia vera (PV) should be excluded.

Elicitation of the absence of *BCR‐ABL1* fusion through real‐time reverse transcriptase polymerase chain reaction (RT‐PCR) or fluorescent in‐situ hybridization (FISH) to determine Philadelphia chromosome rules out CML.^27^ Although, at the time the patient presented to our facility, we could not conduct these tests due to resource limitation. It is however notable that CML would very rarely present with more than 10% monocytes.^5^ Our patient had 24% monocytes, favoring CMML more than CML. Diagnosis of PV and ET requires evidence of hemoglobin (Hb) more than 16 g/dL, platelets more than 450*10^9^/L and the presence of *JAK2*, *CALR* and *MPL* gene mutations on molecular studies.^22^, ^23^, ^28^ Although we were not able to rule out *JAK2*, *CALR* and *MPL* mutations, the presence of low to normal Hb and platelets in our patient seemed to rule out these conditions. Additionally, WHO recommends molecular studies to rule out rare cases of myeloid and lymphoid neoplasms with genetic rearrangements such as *PCM1‐JAK2, PDGFRA, PDGFRB, FGFR1* which would also have eosinophilia and monocytosis in establishing the diagnosis of CMML.^3^, ^4^, ^5^ However, noting lack of significant eosinophilia in our patient, it would be unlikely that he had any of these latter conditions.

Notwithstanding our lack of capacity to conduct molecular studies to rule our CML, ET, PV and other advanced genetic rearrangements that present with eosinophilia and monocytosis, multiparameter flow cytometry may be useful in excluding these differentials, particularly in set ups where molecular studies are not readily available.^29^, ^30^, ^31^ The presence of ≥94% of classical human monocytes which express a high positive CD14 and high negative CD16 surface markers in flow cytometry has a sensitivity and specificity of 91.9% and 94.1% respectively in distinguishing CMML from the aforementioned differentials.^4^, ^26^, ^32^ Notably, our patient expressed 94% CD14 in flow cytometry. Furthermore, our patient also expressed other aberrant cell surface markers such as CD123 (94%) and CD56 (83%) which have been shown to be expressed in CMML.^4^, ^31^

Evidently, diagnosis of CMML is very challenging in low‐resource setting as it requires advance molecular and cytogenetic studies to rule out other MPN or MDS conditions that would present with monocytosis. Noting the different treatment strategies between CMML and other conditions,^21^, ^33^, ^34^ accurate diagnosis of CMML and exclusion of the other causes would be advisable in order to improve outcomes. This may however not be attainable in low‐resource setting noting their limited diagnostic capacity. In our case, the availability of flow cytometry aided the diagnosis, but it is still possible that CML, PV and ET may have been missed due to our lack of capacity to detect mutations that define these conditions. This is therefore a call for different stakeholders in healthcare to improve resource allocation towards the diagnosis and early management of rare hematological malignancies such as CMML.

In conclusion, CMML is a rare hematological disorder, with a highly variable presentation. Its diagnosis requires high index of suspicion especially in low resource setting where advanced diagnostic tools are not readily available. Further, pericardial effusion is a rare but a fatal complication of CMML that clinicians need to be cognisant of. Noting that CMML has a high propensity to transform to AML with poor prognosis, early recognition and initiation of treatment would be highly recommended.

## AUTHOR CONTRIBUTIONS

**Victor M. Wauye:** Conceptualization; data curation; formal analysis; investigation; methodology; project administration; validation; visualization; writing – original draft; writing – review and editing. **Evangeline Njiru:** Conceptualization; investigation; methodology; project administration; supervision; validation; visualization; writing – review and editing. **Angela K. Amadi:** Investigation; methodology; project administration; writing – review and editing. **Mildred N. Hagembe:** Investigation; methodology; project administration; writing – review and editing. **Gabriel Kigen:** Supervision; validation; visualization; writing – review and editing.

## 6 FUNDING INFORMATION

There was no funding obtained for this study.

## ETHICS STATEMENT

Permitted by the Moi University Institutional Review and Ethics Committee, Reference: IREC/723/2023, and approval number: 0004646.

## CONSENT

Written informed consent was obtained from the patient for publication of this case in accordance with the journal's patient consent policy.

## Supporting information


Data S1.

## Data Availability

All data generated or analysed in this study are included in this article and the supporting document.
