# Supplementary material for: Chronic myelomonocytic leukemia primarily presenting as life‐threatening pericardial effusion, Eldoret, Kenya: A case report
Source: Clin Case Rep. 2024 Jun 7;12(6):e9048. doi: 10.1002/ccr3.9048 (PMC11157417; doi:10.1002/ccr3.9048)
Supplement: Supplementary file 1 — Data S1. [file CCR3-12-e9048-s001.docx]

**Case report title:** Chronic Myelomonocytic Leukaemia Primarily Presenting as Life-Threatening Pericardial Effusion: A Case report

**Authors:** Victor M. Wauye, Evangeline Njiru, Angela K. Amadi, Beatrice N. Hagembe, Gabriel Kigen

Laboratory Investigations

|  | | HD1 | HD2 | HD3 | HD4 | HD5 | HD7 | HD8 | HD11 | HD14 | HD15 | HD20 |
| --- | --- | --- | --- | --- | --- | --- | --- | --- | --- | --- | --- | --- |
| CBC | WBC | 103.8 | 75.32 | 74.61 |  | 83.67 | 101.27 |  | 57.66 | 58.14 | 66.4 | 36.92 |
|  | Gra/Ne# | 76.4 | 50.56 | 45.89 |  | 52.09 | 67.07 |  | 34.88 | 30.07 | 34.92 | 18.58 |
|  | Gra/Ne% | 73.4 | 67.6 | 67.1 |  | 61.5 | 62.2 |  | 60.5 | 51.7 | 52.6 | 50.3 |
|  | Lym# | 9.2 | 4.76 | 5.09 |  | 5.25 | 6.83 |  | 4.69 | 6.05 | 6.83 | 7.02 |
|  | Lym% | 8.9 | 6.6 | 6.3 |  | 6.8 | 6.3 |  | 8.1 | 10.4 | 10.3 | 19.0 |
|  | Mon# |  | 18.50 | 22.08 |  | 24.77 | 26.69 |  | 16.93 | 21.11 | 23.94 | 10.44 |
|  | Mon% |  | 24.0 | 24.6 |  | 29.6 | 29.6 |  | 29.4 | 36.3 | 36.0 | 28.3 |
|  | Eos# |  | 1.25 | 1.16 |  | 1.25 | 0.47 |  | 1.00 | 0.75 | 0.52 | 0.71 |
|  | Eos% |  | 1.4 | 1.7 |  | 1.6 | 1.5 |  | 1.7 | 1.3 | 0.8 | 1.9 |
|  | Bas# |  | 0.25 | 0.39 |  | 0.31 | 0.23 |  | 0.16 | 0.16 | 0.19 | 0.17 |
|  | Bas% |  | 0.4 | 0.3 |  | 0.5 | 0.4 |  | 0.3 | 0.3 | 0.3 | 0.5 |
|  | RBC | 3.52 | 3.86 | 3.91 |  | 4.18 | 4.01 |  | 4.11 | 3.62 | 3.67 | 3.41 |
|  | Hb | 11.2 | 12.2 | 12.2 |  | 12.5 | 12.0 |  | 12.8 | 11.3 | 11.5 | 10.6 |
|  | Hct | 31.7 | 34.6 | 35 |  | 37.1 | 35.4 |  | 36 | 31.8 | 32.5 | 29.2 |
|  | MCV | 90.0 | 89.6 | 89.6 |  | 88.6 | 88.2 |  | 87.5 | 87.8 | 88.6 | 85.4 |
|  | MCH | 31.8 | 31.5 | 31.3 |  | 29.9 | 29.9 |  | 31.1 | 31.2 | 31.4 | 30.9 |
|  | RDW | 21.1 | 19.5 | 19.5 |  | 19.3 | 18.8 |  | 19.1 | 18.7 | 18.5 | 17.7 |
|  | Platelets | 167 | 142 | 140 |  | 149 | 171 |  | 138 | 101 | 82 | 35 |
|  | | | | | | | | | | | | |
| UECs | Na^+^ | 135.9 |  | 131 |  | 129 | 136 | 115 | 138 | 136 | 134 |  |
|  | K^+^ | 3.69 |  | 3.74 |  | 3.97 | 5.17 | 4.11 | 3.90 | 3.22 | 3.47 |  |
|  | Cr | 59 |  | 62 |  | 59 | 89 | 67 | 68 | 60 | 80 |  |
|  | U | 19.69 |  | 9.7 |  | 10.4 | 16.5 | 19.0 | 11 | 5.9 | 5.8 |  |
|  | Ca^2+^ | 1.94 |  |  |  | 2.27 | 2.13 | 1.77 | 1.96 | 1.86 |  |  |
|  | Mg^2+^ | 0.69 |  | 0.65 |  |  | 0.81 | 0.69 | 0.56 | 0.50 |  |  |
|  | PO^3-^_4_ |  |  | 0.77 |  |  |  |  |  |  |  |  |
|  | Uric Acid | 615 |  |  |  |  |  |  |  |  |  |  |
|  | | | | | | | | | | | | |
| LFTs | Albumin | 22.27 |  | 23.7 |  |  |  |  |  |  | 27.5 |  |
|  | ALP | 163.5 |  | 164 |  |  |  |  |  |  | 131 |  |
|  | ALT | 19.2 |  | 15.9 |  |  |  |  |  |  | 12.9 |  |
|  | AST | 29.8 |  | 18.2 |  |  |  |  |  |  | 18.5 |  |
|  | T. Bilirubin | 12.5 |  | 15.1 |  |  |  |  |  |  | 20.7 |  |
|  | D. Bilirubin | 6.4 |  | 5.7 |  |  |  |  |  |  | 7.0 |  |
|  | T. Protein | 74.7 |  | 68.6 |  |  |  |  |  |  | 67.2 |  |
|  |  | **HD1** | **HD2** | **HD3** | **HD4** | **HD5** | **HD7** | **HD8** | **HD11** | **HD14** | **HD15** |  |
|  | INR |  |  |  |  |  |  |  |  |  | 3.19 |  |
|  | PT |  |  |  |  |  |  |  |  |  | 32.14 |  |
|  |  |  |  |  |  |  |  |  |  |  |  |  |
| AntiHBsAgAntiHCV HIV status |  | NR |  |  |  |  |  |  |  |  |  |  |
| TFTs | TSH | 1.08 |  |  |  |  |  |  |  |  |  |  |
|  | FT4 | 12.72 |  |  |  |  |  |  |  |  |  |  |
|  | FT3 | 1.41 |  |  |  |  |  |  |  |  |  |  |
| PCT |  |  | 0.572 | 0.748 |  |  | 1.36 |  | 0.567 | 0.342 | 2.76 |  |
| CRP |  |  | 25.85 |  |  |  | 96.30 |  |  |  | 136.97 |  |
| ESR |  | 60 |  |  |  |  |  |  |  |  |  |  |
| PSA |  |  | 3.4 |  |  |  |  |  |  |  |  |  |
| Cardiac Markers | CKMB |  |  | 28.4 |  |  |  |  |  |  |  |  |
|  | CK2 |  |  | 18 |  |  |  |  |  |  |  |  |
|  | TNT |  |  | 23.79 |  |  |  |  |  |  |  |  |
| Urinalysis |  | Hyaline casts 3, Unclassified casts 3, PH 5.5, SG 1.015 | | | | | | | | | | |
|  |  |  |  |  |  |  |  |  |  |  |  |  |
| Pericardial effusion | LDH | 540 |  |  |  |  |  |  |  |  |  |  |
|  | Cholesterol | 1.35 |  |  |  |  |  |  |  |  |  |  |
|  | Albumin | 21.09 |  |  | 19.3 |  |  |  |  |  |  |  |
|  | T. protein | 63.5 |  |  | 51.0 |  |  |  |  |  |  |  |
|  | M/C/S | Nil |  |  |  |  |  |  |  |  |  |  |
| Covid-19 | Rapid Ag |  |  | Neg |  |  |  |  |  |  |  |  |
|  |  |  |  |  |  |  |  |  |  |  |  |  |

HD, Hospital Day
